# VQ-SToRM: Vector-Quantized Smoothness Regularization on Manifolds for Free-Breathing, Ungated Real-Time Cardiac MRI Reconstruction

**DOI:** 10.3390/bioengineering13070764

**Published:** 2026-06-30

**Authors:** Mahrusa Billah, Junpu Hu, Qing Zou

**Affiliations:** 1 Department of Computer Science, The University of Texas at Dallas, Richardson, TX 75080, USA; 2 Department of Biomedical Engineering, The University of Texas Southwestern Medical Center, Dallas, TX 75390, USA; junpu.hu@utsouthwestern.edu; 3 Division of Cardiology, Department of Pediatrics, The University of Texas Southwestern Medical Center, Dallas, TX 75390, USA; 4 Department of Radiology, The University of Texas Southwestern Medical Cente, Dallas, TX 75390, USA; 5 Advanced Imaging Research Center, The University of Texas Southwestern Medical Center, Dallas, TX 75390, USA

**Keywords:** cardiac MRI, real-time imaging, image reconstruction, unsupervised learning, vector quantization, variational autoencoder, discrete latent space, non-Cartesian sampling

## Abstract

Real-time, free-breathing, ungated cardiac magnetic resonance imaging (CMR) is a clinically valuable alternative to conventional breath-held, ECG-gated cine imaging for patients who cannot sustain breath holds or produce reliable cardiac rhythms, including pediatric, arrhythmic, and respiratory-compromised populations. Achieving diagnostic image quality in this setting requires aggressive k-space undersampling and sophisticated reconstruction. Because no fully sampled reference exists for such acquisitions, supervised deep learning is not directly applicable, motivating unsupervised, subject-specific methods. Existing approaches typically rely on low-dimensional continuous latent spaces, which can limit their capacity to represent concurrent cardiac and respiratory motions as distinct states and may suffer from posterior collapse. We introduce VQ-SToRM (Vector-Quantized Smoothness Regularization on Manifolds), an unsupervised framework that adapts the Vector-Quantized Variational Autoencoder to real-time CMR by replacing the continuous latent manifold of prior existing methods with a learned discrete codebook. The encoder, decoder, and codebook are trained jointly on the undersampled non-Cartesian k-t space data of a single subject. On free-breathing, ungated spiral acquisitions from healthy volunteers, VQ-SToRM accurately resolved cardiac and respiratory motion across all phases of the cardiac cycle. A systematic ablation study identified a compact configuration—a codebook of only five embeddings of dimension ten—as optimal, indicating that a small discrete codebook is sufficient to represent the dominant cardiac and respiratory motion content. Compared with V-SToRM and Time-DIP, VQ-SToRM achieved smoother frame-to-frame transitions and comparable or superior signal-to-noise and contrast-to-noise ratios with lower variance across frames and datasets, offering a promising path toward clinically practical real-time CMR.

## 1. Introduction

Cardiac magnetic resonance imaging (CMR) is the gold standard modality for the non-invasive assessment of cardiovascular structure, function, and tissue characterization, offering excellent soft-tissue contrast without ionizing radiation. Standard cine CMR reconstructs cardiac motion by combining data acquired across multiple cardiac cycles, a strategy first popularized by segmented breath-hold-acquisition methods [1] and refined since then through self-gated and parallel-imaging techniques [2,3]. This paradigm relies on electrocardiogram (ECG) gating and breath-holding to suppress motion artifacts. While effective in cooperative adult patients, this paradigm breaks down in several clinically important populations. Pediatric patients, sedated or anesthetized subjects, patients with arrhythmias, and patients with respiratory conditions such as chronic obstructive pulmonary disease or heart failure often cannot sustain breath-holds or produce the regular cardiac rhythm required for reliable gating. In these populations, combining data across cycles introduces inconsistencies that distort the reconstructed motion. Real-time, free-breathing, ungated CMR has therefore emerged as a clinically desirable alternative, offering per-frame temporal resolution without the need for patient cooperation or stable physiology.

Achieving diagnostic-quality real-time CMR, however, requires aggressive k-space undersampling to reach sub-100 ms temporal resolution, which renders the reconstruction problem severely ill-posed. Non-Cartesian trajectories such as radial [4] and spiral [5] readouts are commonly adopted because they oversample the center of k-space and exhibit benign aliasing under undersampling, but they alone are insufficient to recover diagnostic image quality from highly accelerated acquisitions. Compressed sensing methods leveraging spatiotemporal sparsity have partially addressed this gap [6,7,8]. In recent years, deep learning has become the dominant tool not only for accelerated MR reconstruction [9,10,11,12], but also for related fields such as MR segmentation [13] and NMR (nuclear magnetic resonance) CEST (Chemical Exchange Saturation Transfer) [14]. However, supervised deep learning approaches require large training datasets of paired undersampled and fully sampled images [15]—a requirement that is fundamentally incompatible with real-time free-breathing ungated CMR, for which no fully sampled reference exists by construction. This has motivated a growing body of work on unsupervised, subject-specific reconstruction methods that learn directly from the undersampled k-space data of the individual being imaged.

Within this family, generative approaches based on the variational autoencoder (VAE) [16] and deep image prior (DIP) [17] have shown considerable promise. Time-DIP [18] reconstructs dynamic image series by fixing the latent variables and learning only the generator parameters, using shared networks to enforce temporal consistency. Building on the original SToRM framework [19], the Variational Smoothness Regularization on Manifold (V-SToRM) framework [20] extends this idea by modeling time-varying latent vectors as samples from a learned continuous distribution, with a shared CNN generator mapping these vectors to a smooth manifold of cardiac images; the latent vectors capture the intrinsic variability in the dataset, including cardiac and respiratory motion. Related formulations incorporate temporal structure explicitly through Markov models [21,22] or recurrent networks [23]. These methods have established that subject-specific unsupervised reconstruction is a viable path to real-time CMR.

A common design choice across this family of methods, however, is the use of a low-dimensional continuous latent space. While this design offers smoothness and tractability, it can limit the model’s ability to represent the concurrent, quasi-periodic motions present in real-time CMR—cardiac contraction and respiratory displacement—as distinct, well-separated states [24,25]. Continuous VAE-based models are additionally susceptible to posterior collapse, in which the decoder becomes insensitive to the latent code during training [23,26]. These observations motivate exploring alternative latent-space structures that are better suited to representing the discrete phase states inherent to cardiac and respiratory cycles.

The Vector-Quantized Variational Autoencoder (VQ-VAE) [26] replaces the continuous latent space of the standard VAE with a discrete codebook of learned embedding vectors, an architecture that has since been extended for high-fidelity image generation [27]. Encoder outputs are mapped to their nearest codebook entry via vector quantization, and the decoder reconstructs from these discrete codes. This formulation carries three properties that are particularly relevant to real-time CMR. First, the discrete bottleneck eliminates posterior collapse by construction, since each code contributes non-trivially to reconstruction. Second, a higher-dimensional discrete latent space can provide richer expressive capacity than a low-dimensional continuous one. Third, the finite codebook structure may align with the quasi-discrete phase structure of periodic physiology, motivating the hypothesis that a small set of codes could capture the dominant cardiac and respiratory motion content.

In this work, we introduce VQ-SToRM (Vector-Quantized Smoothness Regularization on Manifolds), an unsupervised subject-specific framework that adapts the VQ-VAE architecture to the reconstruction of free-breathing, ungated, real-time cardiac MRI from highly undersampled non-Cartesian k-t space data. Building on the SToRM family of methods, VQ-SToRM replaces the continuous latent manifold with a learned discrete codebook while retaining the shared-network and progressive-training strategies that make subject-specific reconstruction computationally tractable. The encoder, decoder, and codebook are trained jointly on the undersampled measurements of a single subject, with data consistency enforced through the NUFFT and undersampling operator. Our main contributions are as follows:

- To our knowledge, this is the first application of the VQ-VAE architecture to real-time, free-breathing, ungated cardiac MRI reconstruction, extending the SToRM family of unsupervised methods to discrete latent representations.
- Our framework is fully subject-specific: it requires no external training data and learns directly from the undersampled k-space of the subject being imaged, making it immediately applicable to acquisitions for which no ground truth is available.
- Through a systematic ablation study, we show that a compact codebook of only five embeddings is sufficient to represent the dominant cardiac and respiratory motion content of real-time cardiac imaging.
- We compare VQ-SToRM against two state-of-the-art unsupervised methods, V-SToRM and Time-DIP, and demonstrate improved temporal precision with comparable or superior signal-to-noise and contrast-to-noise ratios.

The remainder of this paper is organized as follows. Section 2 details the VQ-SToRM framework, including the vector quantization step and the complete training objective. Section 3 presents the datasets, implementation, ablation study, and comparisons with state-of-the-art methods. Section 4 discusses the implications, limitations, and future directions of this work, and Section 5 concludes.

## 2. Materials and Methods

### 2.1. Problem Formulation

The objective of this work is to reconstruct a time series of MR images $\hat{x}=\left\{{\hat{x}}_{1},{\hat{x}}_{2},\ldots ,{\hat{x}}_{n}\right\}$ of a single short-axis slice of the heart, with high spatiotemporal resolution and cardiac and respiratory motion across *n* frames. Each frame corresponds to undersampled non-Cartesian k-space data $b_{t}$ acquired via sequential spiral readouts at time point *t*. Reconstruction is formulated as the recovery of ${\hat{x}}_{t}$ satisfying

$$\min∥A_{t}{\hat{x}}_{t}-b_{t}{∥}_{2}^{2},$$

where $A_{t}$ is the forward operator at time *t*, combining coil-sensitivity encoding [3], the non-uniform fast Fourier transform (NUFFT) [28], and the undersampling mask associated with the *t*-th set of spiral spokes. We assume that $A_{t}$ satisfies the conditions described in [20] that permit recovery of the true image from its undersampled measurements. Because the reconstruction is severely underdetermined, it must be regularized by a learned prior. We adopt the VQ-VAE as such a prior, as motivated in Section 1, and describe the architecture below.

### 2.2. Encoder, Decoder, and Vector Quantization

The framework consists of three components jointly trained on the undersampled data of a single subject: an encoder $E_{\phi }$ parameterized by ϕ, a decoder $D_{\theta }$ parameterized by θ, and a codebook $C=\{e_{1},e_{2},\ldots ,e_{K}\}$ of *K* learnable embedding vectors, each of dimension *D* [26]. Both $E_{\phi }$ and $D_{\theta }$ are deep convolutional neural networks (CNNs); architectural details are given in Section 3.2.1.

An initial image estimate $x_{t}$ is obtained from the measurements $b_{t}$ via inverse NUFFT with coil combination. The encoder produces an initial latent representation $z_{t}^{e}=E_{\phi }\left(x_{t}\right)\in R^{D}$. This continuous encoder output is then mapped to its nearest codebook entry via vector quantization:

$$z_{t}=e_{k^{*}},\;k^{*}=\arg\min_{j\in \{1,\ldots ,K\}}{∥z_{t}^{e}-e_{j}∥}_{2}.$$

The decoder then reconstructs the MR image from the quantized code: ${\hat{x}}_{t}=D_{\theta }\left(z_{t}\right)$.

Because the argmin operation in the quantization step is non-differentiable, gradients cannot be propagated from the decoder back to the encoder through it directly. We therefore adopt the straight-through estimator (STE) [29]: during the backward pass, the quantization is treated as an identity mapping, so the gradient of the reconstruction loss with respect to the decoder input $z_{t}$ is copied unchanged to the encoder output $z_{t}^{e}$. This approximation is accurate in direction whenever $z_{t}^{e}$ and its assigned embedding $e_{k^{*}}$ are close—a condition that the commitment loss in Equation (1) actively enforces—and in practice it introduces negligible bias while yielding stable convergence without degrading reconstruction quality.

Under this formulation, the prior over latent codes is a discrete uniform distribution, $p(z_{t})=1/K$, and the approximate posterior is one-hot:

$$q\left(z_{t}|x_{t}\right)=\left\{\begin{array}{cc} 1, & z_{t}=\arg\min_{e\in C}{∥e-E_{\phi }\left(x_{t}\right)∥}_{2},\\ 0, & \mathrm{otherwise}. \end{array}\right.$$

Consequently, the Kullback–Leibler divergence between the posterior and the prior, which appears in the standard VAE evidence lower bound, reduces to the constant logK and does not influence optimization [26]. A different training objective, described next, is therefore required.

### 2.3. Training Objective

The VQ-VAE is trained to minimize a three-term loss combining data consistency, codebook learning, and latent-space stability:

(1) $$L_{\mathrm{VQ}}=\underset{\mathrm{data}\;\mathrm{consistency}}{\underset{︸}{∥A\hat{x}{-b∥}_{2}^{2}}}+\underset{\mathrm{codebook}\;\mathrm{loss}}{\underset{︸}{∥\mathrm{sg}\left[z^{e}\right]{-e∥}_{2}^{2}}}+\underset{\mathrm{commitment}\;\mathrm{loss}}{\underset{︸}{\gamma ∥z^{e}{-\mathrm{sg}\left[e\right]∥}_{2}^{2}}},$$

where sg[·] denotes the stop-gradient operator, which blocks gradient flow through its argument during backpropagation [29]. The first term enforces consistency between the k-space from the reconstructed images and the acquired measurements and updates both the encoder parameters ϕ and the decoder parameters θ. The second term, the codebook loss, updates the embedding vectors e so that they track the encoder outputs $z^{e}$. The third term, the commitment loss, updates ϕ so that encoder outputs commit to a chosen embedding rather than drifting between multiple codes; without it, $z^{e}$ can grow unboundedly or oscillate during training [26]. The scalar γ balances these two effects.

**Figure 1:**
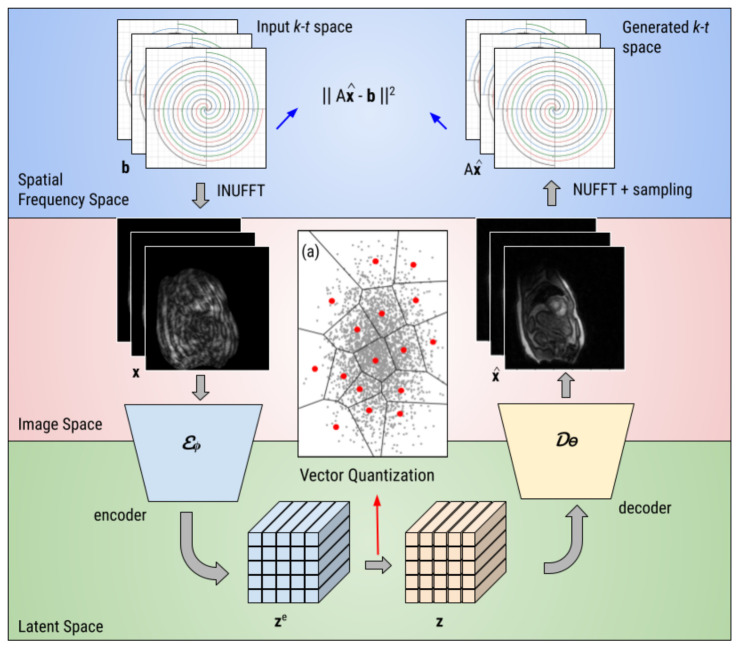
Illustration of the proposed VQ-SToRM framework for real-time cardiac MR image reconstruction. Starting from the top-left and moving counterclockwise, k-t space data b is acquired using non-Cartesian spiral readouts. Following coil combination and inverse NUFFT, the input image time series x is passed through the encoder CNN $E_{\phi }$ to produce initial latent vectors $z^{e}$. Each $z^{e}$ undergoes vector quantization (VQ), illustrated as a Voronoi partition in which each cell contains one embedding vector $e_{i}$ (red circles) and a set of encoder outputs (gray dots); all encoder outputs within a cell are replaced by the corresponding embedding. The quantized codes are then passed through the decoder CNN $D_{\theta }$ to produce the reconstructed image series $\hat{x}$. Applying the forward operator *A* (combining coil encoding, NUFFT, and undersampling) yields the reconstructed k-space, which is compared against b via the data-consistency term of Equation (1).

In addition to $L_{\mathrm{VQ}}$, we include a squared-$l_{1}$ regularization on the decoder parameters to stabilize training, following [30]. The complete training objective is

(2) $$L=L_{\mathrm{VQ}}+\lambda {∥\theta ∥}_{1}^{2},$$

where λ is a weighting coefficient set empirically. The model is trained using the progressive-in-time scheme of [30], in which the temporal resolution of the reconstructed series is increased in stages to reduce computational cost while stabilizing optimization. Figure 1 illustrates the overall framework, showing the flow of data between the k-t space, image domain, and discrete latent space.

## 3. Results

### 3.1. Datasets and Preprocessing

Fourteen slices of 2D real-time cardiac MR data were acquired from two healthy adult volunteers on a 3T MR scanner using a golden-angle spiral trajectory in the short-axis view under free-breathing conditions and without electrocardiogram gating or chest-displacement gating. The data acquisition was approved by the local Institutional Review Board (IRB), and consent forms were obtained from both participants. Each acquisition was compressed into eight virtual coil arrays, with 2336 k-space points per spoke. Coil sensitivity maps for each slice were estimated using ESPIRiT [31] from all k-space data corresponding to that slice. Each subject’s data was reorganized into a time series of 150 frames, each reconstructed from six sequential spirals and corresponding to a temporal footprint of 50 ms. The reconstructed image matrix is 340×340.

### 3.2. Implementation Details

#### 3.2.1. Network Architecture

The encoder and decoder of our VQ-SToRM framework are deep CNNs. The encoder consists of six convolutional layers, each followed by a leaky ReLU activation [32], and maps a two-channel (real and imaginary) input image to a latent representation with d·4 channels, where *d* is a user-defined network-width parameter. The decoder consists of seven transpose-convolutional layers, with leaky ReLU activations between layers and a tanh activation following the final layer. While the original VQ-VAE formulation [26] suggests matching the embedding dimension to the number of encoder output channels, we found that a substantially smaller embedding dimension reduced reconstruction error, consistent with prior observations that VQ-VAEs tend to compress higher-dimensional embeddings into a lower-dimensional subspace [33]. The ablation study described in Section 3.3 identifies d=60, embedding dimension D=10, and codebook size K=5 as the optimal configuration. Under this configuration, the model contains approximately 13.6 million trainable parameters, roughly half the size of the reconstructed image series volume. We adopt this configuration for all comparisons with state-of-the-art methods in Section 3.4.

#### 3.2.2. Training Settings

The model is trained using the progressive-in-time scheme of [30], in which the temporal resolution of the reconstructed series is increased in stages. In the first stage, the k-t space data is divided into ten average frames and the model is trained for 1000 epochs at learning rates of $5\times 10^{-5}$ for the encoder and $1\times 10^{-4}$ for the decoder. In the second stage, the same k-t space is divided into the full temporal resolution of 150 frames, and the model is trained for an additional 1800 epochs, with learning rates of $2\times 10^{-5}$ for the encoder and $1\times 10^{-4}$ for the decoder. Optimization is performed using the Adam optimizer [34]. The commitment-loss coefficient γ in Equation (1) is set to 0.25, following the default value in [26]. All training was performed on a single NVIDIA RTX 4500 Ada Generation GPU. For a subject-specific method of this kind, the optimization is itself the reconstruction: training the network on a given acquisition directly produces the reconstructed image series, and the resulting model is specific to that dataset. Under the final configuration, this complete two-stage optimization required approximately 10 min per dataset on the above hardware.

### 3.3. Ablation Study

We performed a systematic ablation study to identify the hyperparameters that minimize reconstruction loss while preserving spatiotemporal image quality. Starting from an initial configuration of d=52, D=100, K=150, we adopted a sequential protocol: first sweeping the network width *d* (Section 3.3.1), then seeping the embedding dimension *D* at the chosen *d* (Section 3.3.2), and finally performing a joint sweep over (D,K) to determine the codebook configuration (Section 3.3.3). Reconstruction loss refers to the data-consistency term $∥A\hat{x}{-b∥}_{2}^{2}$ evaluated at convergence.

#### 3.3.1. Network Width

**Figure 2:**
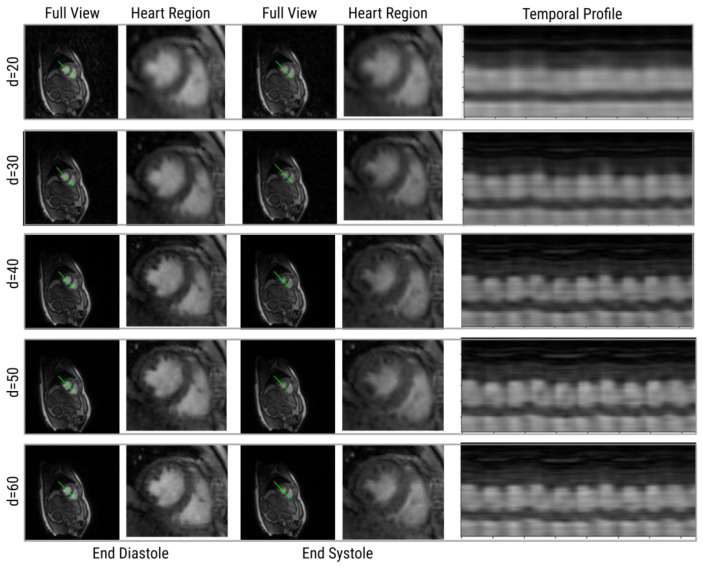
Effect of network width *d* on reconstruction performance. Representative results are shown for d∈{20,30,40,50,60}. For each configuration, the figure displays diastolic and systolic reference frames alongside the temporal profile at the green line across the 150-frame series.

The user-defined parameter *d* controls the number of convolutional filters in both the encoder and decoder and therefore the total number of trainable parameters in the model. These experiments were performed at the initial embedding configuration (D=100, K=150) with d∈{20,30,40,50,60}; representative results are shown in Figure 2. All configurations captured the respiratory motion of the subject. However, at d=20 the network failed to accurately depict cardiac motion and did not resolve fine structures such as the papillary muscles. At d=30, cardiac motion was partially recovered, but the myocardium and papillary muscles remained poorly delineated. For d∈{40,50,60}, the scheme reconstructed the image series with high spatiotemporal resolution and accurately depicted both respiratory and cardiac motion, with d=60 yielding the highest contrast and lowest reconstruction loss. Because *d* primarily controls network capacity rather than the geometry of the latent space, its optimum is expected to be largely decoupled from the choice of *D* and *K*. We therefore fix d=60 for all subsequent ablations and for the comparisons with state-of-the-art methods.

#### 3.3.2. Embedding Dimension

**Figure 3:**
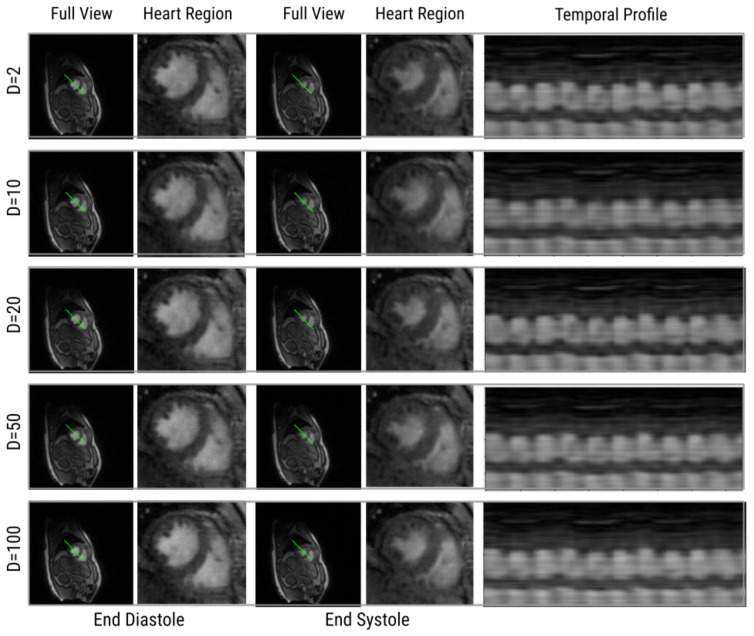
Effect of embedding dimension *D* on reconstruction performance. Representative results are shown for D∈{2,10,20,50,100}, all at d=60 and K=150. For each configuration, the figure displays diastolic and systolic reference frames alongside the temporal profile at the green line across the 150-frame series.

With d=60 fixed, we next investigated the effect of the embedding dimension *D* on reconstruction performance, holding the codebook size at the initial value K=150. We tested D∈{1,2,10,20,50,75,100,150}; a representative subset is shown in Figure 3. The model captured both respiratory and cardiac motion at all tested dimensions, with only small differences in image quality, temporal resolution, and contrast. The lowest reconstruction loss in this single-parameter sweep occurred at D=2, consistent with prior observations that VQ-VAEs often favor lower-dimensional representations [33,35]. However, several larger dimensions (for example D=20 and D=50) achieved lower loss than D=10, indicating that the relationship between *D* and reconstruction quality is not monotonic at fixed *K*. This non-monotonicity motivated the joint sweep over (D,K) described in Section 3.3.3.

#### 3.3.3. Codebook Size

**Figure 4:**
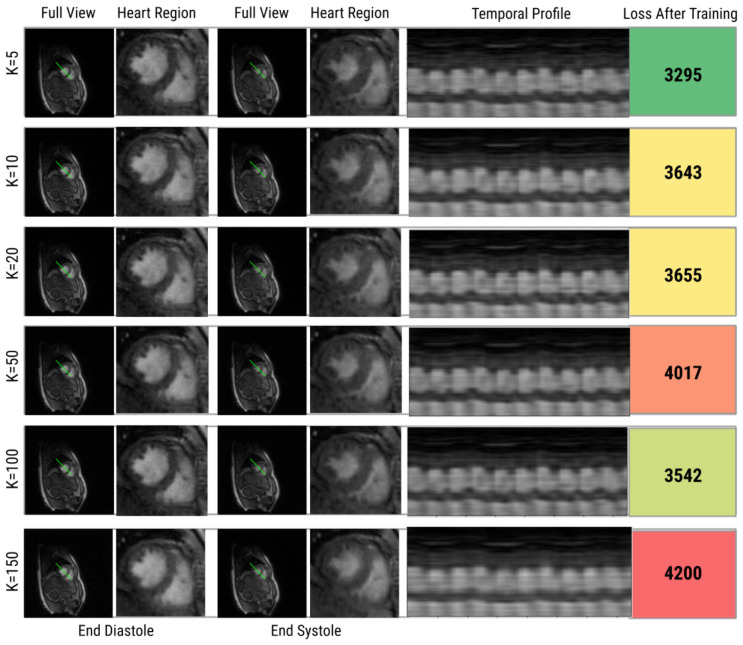
Effect of codebook size *K* on reconstruction performance at D=10. Representative results are shown for K∈{5,10,20,50,100,150}, all at d=60. For each configuration, the figure displays diastolic and systolic reference frames alongside the temporal profile at the green line across the 150-frame series. The lowest reconstruction loss was achieved at K=5.

Finally, we investigated the codebook size *K* jointly with the embedding dimension *D*. While a larger codebook may in principle allow finer temporal discrimination, excessively large codebooks can lead to over-fragmentation of the latent space [36]. Motivated by the single-parameter *D*-sweep, we focused on the two most promising embedding dimensions, D=2 and D=10, and varied K∈{1,5,10,20,50,100,150}, yielding 14 joint configurations. For all configurations, the framework successfully captured cardiac and respiratory motion with satisfactory resolution across the 150-frame series. Among these, the lowest reconstruction loss was achieved at D=10, K=5, which also outperformed the best single-parameter configuration D=2, K=150. This choice aligns with the motivation presented in Section 1: a higher-dimensional (D≥3) discrete latent space provides richer expressive capacity than a low-dimensional continuous one, while a compact codebook (K=5) is sufficient to represent the dominant cardiac and respiratory motion content. We note, however, that a direct correspondence between individual codebook entries and specific physiological phases was not borne out by subsequent inspection of the learned codes (Section 4). Figure 4 shows results for the D=10 sweep. We adopt d=60, D=10, K=5 as the final configuration for all subsequent experiments.

### 3.4. Comparison with State-of-the-Art Methods

We compared VQ-SToRM against two recent unsupervised, subject-specific methods for free-breathing ungated real-time CMR reconstruction:

- V-SToRM [20]: A VAE-based method that models time-varying latent vectors as samples from a learned continuous distribution on a smooth manifold. A shared CNN generator maps these latents to reconstructed image frames, and model parameters are estimated from the undersampled measurements via backpropagation. The continuous latent vectors are intended to capture the intrinsic variability of the dataset, including cardiac and respiratory motion. The model has about 4 million parameters. We selected this method for comparison, as V-SToRM represents the most relevant prior work in this line of research and directly motivated the development of VQ-SToRM. Comparing against V-SToRM provides the clearest assessment of the advances introduced by the proposed method.
- Time-DIP [18]: a deep-image-prior approach in which the latent variables are fixed rather than learned, and only the generator parameters are optimized. For real-time applications, Time-DIP fixes a period—here set to 20 frames, approximately the cardiac cycle duration in the dataset—and draws the latent vectors for frames at multiples of that period as independent Gaussian samples, with the intermediate frames interpolated linearly. The model has 4.4 million parameters. This method was chosen for comparison because it is a strong and widely used state-of-the-art method and shares key design characteristics with VQ-SToRM and V-SToRM.

We restricted our comparison to unsupervised, subject-specific methods that, like VQ-SToRM, reconstruct directly from the undersampled k-space of a single subject without any external training data. Other widely used dynamic-MRI reconstruction methods are not directly comparable in this setting. Supervised deep-learning approaches such as MoDL [10] and recent transformer-based networks require large datasets of paired undersampled and fully sampled images, which by construction do not exist for free-breathing, ungated, real-time acquisitions. Compressed-sensing methods such as XD-GRASP [8] rely on binning the acquired data into discrete cardiac and respiratory motion states (extra-dimensional sorting), which presupposes the gating information that is precisely what is unavailable in the ungated real-time regime targeted here. V-SToRM and Time-DIP were therefore selected as the most relevant and directly comparable state-of-the-art baselines.

All three methods (VQ-SToRM, V-SToRM, and Time-DIP) were evaluated on all datasets described in Section 3.1. We present qualitative and quantitative comparisons below.

#### 3.4.1. Qualitative Comparison

**Figure 5:**
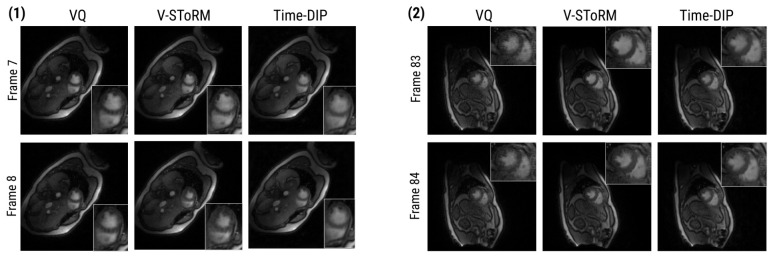
Qualitative comparison between VQ-SToRM, V-SToRM [20], and Time-DIP [18]. For each method, the figure shows full-view and zoomed-in heart regions for two consecutive frames (50 ms apart), for datasets (**1**,**2**).

Figure 5 compares representative frames from the 150-frame image series reconstructed by each method. We show two consecutive frames, 50 ms apart, reconstructed by each method, to evaluate both per-frame image quality and frame-to-frame temporal consistency. Time-DIP exhibits reduced contrast between the blood pool and the myocardium and does not clearly delineate the papillary muscles; it also shows little detectable change between the two consecutive frames, indicating limited temporal sensitivity. VQ-SToRM and V-SToRM produce visually comparable per-frame image quality and contrast. Between the two consecutive frames, V-SToRM depicts an abrupt change in ventricular volume, whereas VQ-SToRM depicts a more gradual but still clearly visible change—consistent with the expected smooth evolution of cardiac motion at 50 ms intervals.

**Figure 6:**
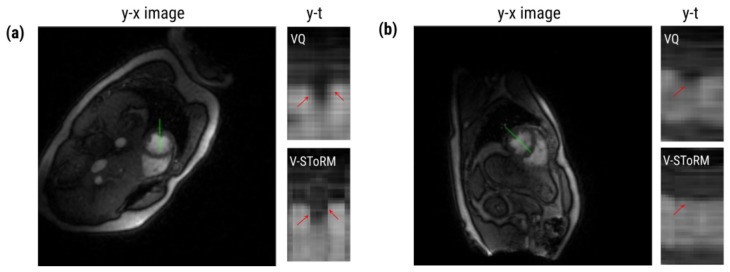
Temporal profiles through the heart region for image series reconstructed by VQ-SToRM and V-SToRM, for two cardiac slice positions (**a**,**b**), over approximately 20 consecutive frames. (**Left**): reference frame with the profile line highlighted in green. (**Right-upper**): VQ-SToRM time profile. (**Right-lower**): V-SToRM time profile. Red arrows indicate locations where VQ-SToRM produces smoother frame-to-frame transitions than V-SToRM.

Figure 6 shows y–t temporal profiles through the heart region for VQ-SToRM and V-SToRM over approximately 20 consecutive frames, at two different slice positions. The time profiles from VQ-SToRM show smooth, gradual transitions between cardiac phases across the series. V-SToRM produces broadly similar profiles but with more abrupt transitions between some adjacent frames (red arrows). This is consistent with the observations in Figure 5 and supports the interpretation that a discrete latent space can represent the progression between cardiac phases as a smoother sequence of codebook transitions than a continuous latent space tends to produce in practice.

#### 3.4.2. Quantitative Comparison

**Figure 7:**
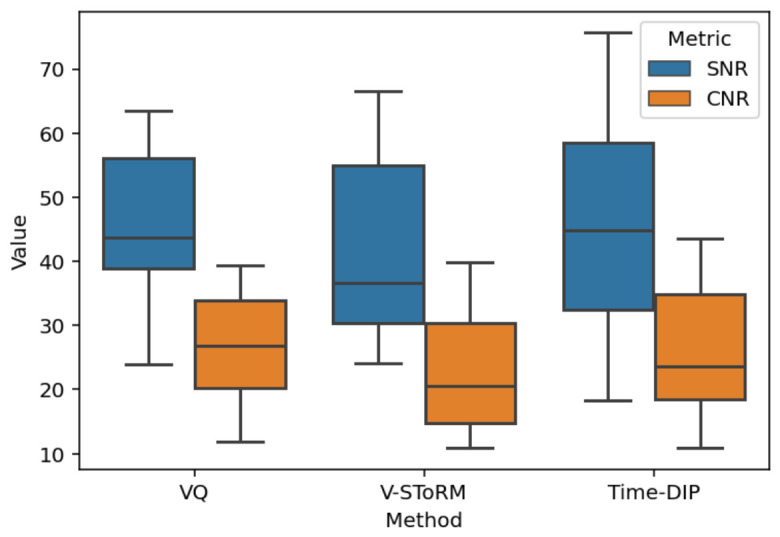
Box-plot comparison of SNR and CNR between VQ-SToRM, V-SToRM, and Time-DIP, pooled across frames from both datasets.

We evaluated all three methods using signal-to-noise ratio (SNR) and contrast-to-noise ratio (CNR), two metrics commonly used to assess CMR image quality. SNR was computed as

$$\mathrm{SNR}=\frac{\mu_{b}}{\sigma_{n}},$$

where $\mu_{b}$ is the mean signal intensity within a region of interest in the blood pool and $\sigma_{n}$ is the standard deviation of signal intensity in a noise region of interest placed in the lung parenchyma. CNR was computed as

$$\mathrm{CNR}=\frac{\mu_{b}-\mu_{m}}{\sigma_{n}},$$

where $\mu_{m}$ is the mean signal intensity within a region of interest in the myocardium. SNR and CNR were computed per frame and pooled across frames and datasets. The resulting distributions are summarized in Figure 7 and Table 1.

VQ-SToRM produced SNR and CNR values that were comparable to or higher than those of the two baseline methods. VQ-SToRM achieved the highest median CNR among the three methods and the smallest rightward skew in the CNR distribution. VQ-SToRM also exhibited lower variance in both SNR and CNR than V-SToRM and Time-DIP, indicating more consistent image quality across frames and datasets. Taken together with the qualitative observations in Section 3.4.1, these results indicate that VQ-SToRM improves temporal precision in real-time CMR reconstruction without compromising per-frame image quality.

### 3.5. Reconstruction Showcase

**Figure 8:**
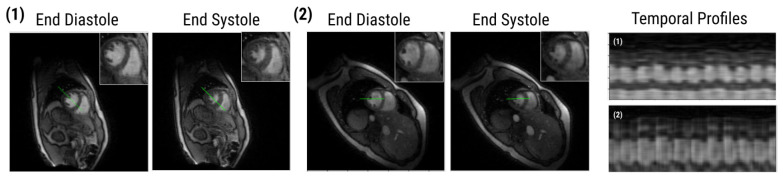
Representative VQ-SToRM reconstructions at the final configuration (d=60, D=10, K=5). (**Left**): full-view and zoomed-in heart regions at end-diastole and end-systole for datasets (**1**,**2**). (**Right**): temporal profiles across the 150-frame series for datasets (**1**) (**top**) and (**2**) (**bottom**).

To illustrate the end-to-end behavior of VQ-SToRM under the final configuration (d=60, D=10, K=5), Figure 8 shows representative reconstructions and temporal profiles for two datasets. VQ-SToRM resolves all phases of the cardiac cycle, from end-diastole to end-systole, and clearly depicts the concurrent respiratory motion in the time profiles. Transitions between adjacent frames are smooth without loss of cardiac-phase detail, and the reconstructions maintain SNR and CNR within the ranges reported in Section 3.4.2. The shared-encoder and shared-decoder design, combined with the compact discrete codebook, thus enables accurate depiction of both cardiac and respiratory motion from highly undersampled free-breathing, ungated acquisitions.

### 3.6. Codebook Utilization

While the discrete bottleneck of the VQ-VAE eliminates posterior collapse by construction, it does not preclude codebook collapse, a distinct failure mode in which a subset of embeddings is never (or only rarely) selected and the effective codebook size shrinks below *K* [36]. This risk is particularly relevant for small codebooks such as the K=5 configuration adopted here. To assess it, we examined the distribution of code assignments across the 150-frame series at the final configuration. We found that all five embeddings were actively used, with selection counts of 22.7%, 20.7%, 14.7%, 18.0%, and 24.0% for codes 0–4, respectively. No embedding was left unused, and the most frequently selected entry was chosen only about 1.6 times as often as the least frequently selected one, indicating a fairly balanced and fully utilized codebook with no evidence of codebook collapse.

## 4. Discussion

In this work, we introduced VQ-SToRM, an unsupervised subject-specific framework that adapts the VQ-VAE architecture to real-time, free-breathing, ungated cardiac MRI reconstruction. The framework replaces the continuous latent manifold of prior SToRM-family methods with a learned discrete codebook, and is trained jointly with the encoder and decoder networks on the undersampled k-space measurements of a single subject.

A central finding of our ablation study is that a very compact codebook—only five embeddings of dimension ten—minimizes reconstruction loss across the 150-frame series, outperforming substantially larger codebooks. We interpret this as evidence that the discrete latent space has learned a parsimonious representation of the dominant motion states in real-time CMR. Because cardiac and respiratory motion are quasi-periodic and composed of a small number of canonical phases (for example, end-diastole, peak systole, and the inspiratory and expiratory extrema of the respiratory cycle), a small codebook is sufficient to parameterize the manifold of reconstructed images. We emphasize, however, that this should be understood at the level of the codebook as a whole rather than as a strict one-to-one mapping: as detailed in the limitations below, an explicit inspection of the learned codes did not reveal a strong correspondence between individual codebook entries and specific cardiac or respiratory phases. The decoder then interpolates between these discrete codes to produce the full image series. This interpretation is consistent with the observation that a higher-dimensional continuous latent space is not required to achieve competitive reconstruction quality: what matters is not the dimensionality of the latent code but its ability to separate distinct motion states, which the discrete codebook achieves by construction.

Beyond the empirical ablation, the selected configuration admits a theoretical interpretation grounded in the structure of the reconstruction problem and the known behavior of vector-quantized models. From a rate–distortion perspective, the codebook size *K* sets the rate of the discrete bottleneck: it upper-bounds the number of distinguishable states the decoder can receive, so the smallest *K* that still spans the dominant motion states minimizes distortion without allocating capacity to noise. Because free-breathing cardiac dynamics are governed by two quasi-periodic processes—cardiac contraction and respiration—the intrinsic dimensionality of the underlying motion manifold is low, and a small number of canonical phase combinations suffices to parameterize it, consistent with the observed optimum at K=5. The choice of a comparatively small embedding dimension *D* is likewise consistent with the dimension-collapse phenomenon reported for VQ-VAEs, in which only a low-dimensional subspace of the embedding space is effectively used [33,35]; setting *D* near this effective dimensionality avoids wasted capacity and the optimization difficulty of quantizing in a high-dimensional space. Conversely, excessively large *K* or *D* fragment the latent space and reduce the number of frames assigned to each code, weakening the implicit regularization that stabilizes the reconstruction. These arguments provide a principled rationale for the empirically selected configuration, although a complete theoretical characterization of the optimum remains an open question.

A potential concern is that the encoder operates on inverse-NUFFT image estimates that contain undersampling artifacts, which could in principle lead the model to encode artifact-related features rather than genuine physiological motion. Several aspects of our formulation mitigate this. The training objective is driven by data consistency against the acquired k-space measurements rather than by reproducing the artifact-laden encoder input, so the network is not rewarded for preserving aliasing artifacts. Moreover, undersampling artifacts arising from the golden-angle spiral trajectory are temporally incoherent across frames, whereas cardiac and respiratory motion are structured and quasi-periodic; the compact discrete codebook, which must explain all 150 frames with only five embeddings, therefore preferentially captures the dominant, temporally consistent motion states rather than frame-specific artifacts. The accurate depiction of cardiac and respiratory motion and the absence of residual streaking in the reconstructions (Figure 5, Figure 6, Figure 7 and Figure 8) are consistent with this interpretation.

The clinical motivation for real-time, free-breathing, ungated CMR lies in the patient populations for whom breath-holding and reliable ECG gating are difficult or impossible: pediatric patients, sedated or anesthetized subjects, patients with arrhythmias, and patients with respiratory or cardiac conditions that preclude prolonged breath-holds. By eliminating the need for external training data and reconstructing from highly undersampled acquisitions, VQ-SToRM is directly applicable to these populations: each reconstruction uses only the k-space data of the subject being imaged, removing the distributional-shift concerns that affect supervised methods trained on adult, breath-held, gated datasets. The compact codebook may also offer an avenue for interpretability of potential clinical interest: should individual codebook entries be found to correspond reliably to identifiable cardiac or respiratory phases—which, as noted below, was not the case in our present analysis—they could potentially be used for retrospective gating or phase-based analysis without additional physiological monitoring, in the spirit of self-gated CMR techniques that derive motion signals directly from the acquired data [2]. We consider this a promising direction for future work but beyond the scope of the present study.

Several limitations of this study should be acknowledged. First, the evaluation was performed on two healthy adult volunteers, which is appropriate for a proof-of-concept methodological study but does not support generalization claims. Validation on a larger and clinically more diverse cohort—including pediatric patients, patients with arrhythmias, and patients with structural heart disease such as single-ventricle physiology—is required before VQ-SToRM can be considered clinically deployable. Validation on public datasets, while desirable for benchmarking, is currently constrained by the scarcity of suitable data: to our knowledge, publicly available cardiac MR datasets are predominantly Cartesian and acquired under breath-held, ECG-gated protocols, whereas VQ-SToRM targets free-breathing, ungated, real-time non-Cartesian (spiral) acquisitions, for which raw k-space data are not yet publicly released. We further note that some suggested benchmarks, such as LLD-MMRI, comprise reconstructed multi-phase liver MR images intended for lesion diagnosis rather than raw cardiac k-space measurements, and are therefore not directly applicable to the reconstruction task addressed here. Validating the method on larger, clinically diverse cardiac cohorts and on emerging public real-time datasets as they become available is an important direction for future work. Second, in the absence of a fully sampled reference (which cannot be obtained for free-breathing, ungated real-time acquisitions by construction), our quantitative evaluation relies on SNR and CNR rather than on reference-based metrics such as normalized root-mean-squared error or the structural similarity index [37]. Developing appropriate reference-free metrics for real-time CMR remains an open problem in the field. Additionally, future work could incorporate Local Binary Pattern (LBP)-based texture loss as an auxiliary perceptual loss. This could better preserve fine myocardial structures and subtle pathologies [38]. Third, as a subject-specific method, VQ-SToRM requires training a new model for each acquisition, which carries a higher computational cost than pretrained supervised networks that perform inference in a single forward pass. We clarify, however, that the term real-time here refers to the acquisition regime—capturing cardiac and respiratory dynamics at high temporal resolution without ECG gating or breath-holding—rather than to an online reconstruction-speed requirement. As with other subject-specific unsupervised methods such as V-SToRM and Time-DIP, reconstruction is performed retrospectively (offline) rather than concurrently with acquisition: the network optimization is itself the reconstruction step, requiring approximately 10 min per dataset on a single NVIDIA RTX 4500 Ada GPU. This one-time, per-acquisition cost is acceptable for offline processing and does not need to keep pace with the scanner, while the absence of any external training requirement avoids large-scale data collection and the distributional-shift concerns of supervised methods. Reducing this training cost—through architectural simplifications, transfer from pre-trained codebooks, or hybrid schemes that combine subject-specific fine-tuning with population-level priors—is an important direction for future work. Concurrently, upgrading the plain CNN to a deeply connected architecture (e.g., DenseNet-121) could mitigate vanishing gradients and reduce overfitting during subject-specific training, drawing on deep encoding frameworks proven in other constrained learning tasks [39]. Finally, although the compactness of the optimal codebook is suggestive of an underlying low-dimensional motion structure, we caution against over-interpreting individual codebook entries as discrete physiological phase states. An explicit inspection of the learned codes—examining which frames were assigned to which codes and whether those assignments aligned with cardiac or respiratory phase—did not reveal a strong or consistent correspondence between individual entries and specific physiological phases. We therefore refrain from claiming a direct codebook-to-physiology mapping; characterizing the conditions under which such an interpretable correspondence might emerge, for example, through codebook-usage regularization or explicit physiological guidance, remains an interesting direction for future work.

## 5. Conclusions

We presented VQ-SToRM, an unsupervised subject-specific framework that adapts the vector-quantized variational autoencoder to the reconstruction of free-breathing, ungated, real-time cardiac MRI from highly undersampled non-Cartesian k-t space data. By replacing the continuous latent manifold of prior SToRM-family methods with a learned discrete codebook, VQ-SToRM avoids posterior collapse by construction and provides a parsimonious representation of the dominant cardiac and respiratory motion states. Across the experiments reported here, a codebook of only five embeddings was sufficient to reconstruct 150-frame image series with accurate depiction of both cardiac and respiratory motion. In comparison with two state-of-the-art unsupervised methods, VQ-SToRM achieved smoother frame-to-frame transitions and comparable or improved signal-to-noise and contrast-to-noise ratios, with lower variance across frames and datasets. These findings position discrete latent representations as a promising direction within the unsupervised CMR reconstruction literature and motivate further evaluation in clinically diverse cohorts.

## Figures and Tables

**Table 1:** Comparison of average SNR and CNR between VQ-SToRM, V-SToRM, and Time-DIP, pooled across frames from both datasets. Best results are highlighted in blod.

	Time-DIP	V-SToRM	VQ-SToRM
SNR (mean)	45.41	40.55	**46.28**
CNR (mean)	25.72	22.98	**27.02**

## Data Availability

The data presented in this study are available on reasonable request from the corresponding author. The data are not publicly available due to privacy restrictions associated with human subject imaging data.

## Abbreviations

The following abbreviations are used in this manuscript:

CEST	Chemical Exchange Saturation Transfer
CMR	Cardiac Magnetic Resonance
CNN	Convolutional Neural Network
CNR	Contrast-to-Noise Ratio
DIP	Deep Image Prior
ECG	Electrocardiogram
ELBO	Evidence Lower Bound
ESPIRiT	Eigenvalue-based iterative Self-consistent Parallel Imaging Reconstruction
KL	Kullback–Leibler (divergence)
LBP	Local Binary Pattern
NMR	Nuclear Magnetic Resonance
NUFFT	Non-Uniform Fast Fourier Transform
SENSE	Sensitivity Encoding
SNR	Signal-to-Noise Ratio
SToRM	Smoothness Regularization on Manifolds
VAE	Variational Autoencoder
VQ-VAE	Vector-Quantized Variational Autoencoder
VQ-SToRM	Vector-Quantized Smoothness Regularization on Manifolds
